# 

**Published:** 1912-06

**Authors:** 


					﻿Vol. XXVII
*5uNE; 1912
No. 12
B I T E A S
MHIDICa..
JOURNA1]
Established
July, 1885
“Red Back”
PUBLISHED MONTHLY AT AUSTIN. TEXAS. BY
F. E. DANIEL., M. D., - - - Editor and Proprietor.
PRINTED BY THE VON BOECKMANN-JONES CO.
Subscription, $1.00 a Year, in Advance. -	-	-	- Single Copies, 10 Cents
Entered at the Postoffice at Austin. Texas, as second class mail matter.
				

